# Editorial: Sleep Deprivation, Circadian Misalignment and Addiction Vulnerability in Adolescents

**DOI:** 10.3389/fnins.2022.940039

**Published:** 2022-07-06

**Authors:** Wen-Jie Bian, Fatin Atrooz, Sanket Patel, Abeer M. Rababa'h

**Affiliations:** ^1^Department of Psychiatry and Behavioral Sciences, Stanford University School of Medicine, Stanford, CA, United States; ^2^Wu Tsai Neurosciences Institute, Stanford University, Stanford, CA, United States; ^3^Department of Pharmacological and Pharmaceutical Sciences, College of Pharmacy, University of Houston, Houston, TX, United States; ^4^Department of Clinical Pharmacy, College of Pharmacy, Jordan University of Science and Technology, Irbid, Jordan

**Keywords:** sleep deprivation, circadian misalignment, addiction, neurodevelopmental disorders, substance use disorders, adolescent development

Sleep is essential for almost all organisms, and it demonstrates a clear developmental trajectory in the amount of sleep needed from the early postnatal stage to adulthood (Hirshkowitz et al., 2015). The National Sleep Foundation of the United States of America recommends a minimum of ~8 h of daily sleep for adolescents (Hirshkowitz et al., 2015). However, a modern lifestyle involving excessive use of electronic devices, school/work, active social life, and late-night activities may have led to prevalent sleep problems including delayed sleep onset, shortened sleep duration, sleep fragmentation, and social jetlag in adolescents all around the world (Gariepy et al., 2020; Wheaton and Claussen, 2021), which are further impacted by a stressful life event such as the COVID-19 pandemic (Becker et al., 2021). Adolescence is a critical developmental stage when the brain undergoes substantial circuit refinement and molecular changes important for its maturation (Casey et al., 2008; Moyer and Zuo, 2018; Faust et al., 2021). Thus, the adolescent brain is likely to be more vulnerable, compared to the adult brain, to sleep disturbances, circadian rhythm disruption, and associated substance abuse. This Research Topic highlights the developmental role of sleep and the interconnections of sleep regulation, circadian rhythm, and the neural circuits involved in addiction and reward-seeking behavior, and gathers several contributions, including three original research articles and a systematic review, from studies in different species using different approaches.

One of the articles of this Research Topic (Hasler et al.) reported a human study in 31 late adolescent females (18–22 y/o) assessing the neural response to monetary reward in correlation with the circadian alignment of sleep and weekend drinking. The authors showed that greater circadian misalignment prior to the weekend, assessed via the dim light melatonin onset (DLMO), was associated with lower neural responses in the mesocorticolimbic reward pathway to anticipated reward as measured by functional magnetic resonance imaging (fMRI), which further correlated with more binge-drinking during the weekend. They further showed a positive link between the weekend social jetlag and post-weekend neural responses to anticipated rewards. The study provided preliminary evidence for the association between sleep/circadian disruption and the activity of neural circuits underlying reward signaling in human adolescent subjects.

Using a rodent model, the causal link between early-life sleep deprivation and reward-seeking behavior in adolescence was further interrogated by Atrooz et al.. The authors adopted a 14-day sleep deprivation protocol using a rotating bar setup during the pre-adolescent period from postnatal day (PND) 19 to PND32 in rats. After the conclusion of sleep deprivation protocol, at PND33, the authors examined the rats for anxiety-like and depression-like behaviors using the elevated plus maze test and the sucrose splash test, respectively. They also assessed alcohol consumption in rats at the adolescent stage starting at PND39 for five consecutive days using voluntary alcohol drinking test. They found that compared to the control rats, the sleep deprived (SD) animals spent significantly less time in the open arm in the elevated plus-maze test and reduced grooming in the sucrose splash test, suggesting increased anxiety- and depression-like behaviors. When the animals grew to become adolescents, interestingly, SD rats showed significantly increased consumption of alcohol together with an elevated alcohol preference over water as compared to control rats, likely due to prior sleep deprivation.

As suggested by these two intriguing studies, adequate sleep during development is believed to play a critical role in shaping important brain functions. Emerging evidence has revealed that persistent sleep disturbances during early life developmental stages (e.g., early childhood, adolescence) are likely to lead to negative consequences including substance use and abuse, and under pathological conditions may contribute to the progression of mental illnesses. Alrousan and colleagues further reviewed a large body of literature in Alrousan et al. and discussed the link between sleep and postnatal brain development, the molecular mechanisms that might be involved, and the available animal models based on knowledge and insights gained from recent human and animal studies.

What are the structural underpinnings of sleep's developmental role? In an attempt to tackle this question, Brodin et al. compared the dendrite and spine morphology of hippocampal CA1 pyramidal neurons in adult mice after acute single sleep deprivation challenge with that in the control mice, using the tissue-clearing technique, See Ke et al., 2016. However, the authors found no significant change in the dendritic arborization or spine density/morphology in the CA1 pyramidal neurons. The authors suggested that a single episode of sleep deprivation has no major neuroanatomical changes. On the other hand, given the previous studies demonstrating that sleep does regulate the dynamics and ultrastructure of cortical and hippocampal spines in juvenile mice (Maret et al., 2011; Yang et al., 2014; de Vivo et al., 2017; Li et al., 2017; Spano et al., 2019; Zhou et al., 2020; Nagai et al., 2021), it is also likely that the impact of sleep deprivation on dendritic spines is more profound during development than in adulthood.

We hope that this Research Topic brings intriguing insights to our readers regarding the impact of sleep deprivation during developmental stages on reward seeking behavior and mental health of adolescents.

## Author Contributions

W-JB wrote the manuscript with contributions from FA, SP, and AR.

## Conflict of Interest

The authors declare that the research was conducted in the absence of any commercial or financial relationships that could be construed as a potential conflict of interest.

## Publisher's Note

All claims expressed in this article are solely those of the authors and do not necessarily represent those of their affiliated organizations, or those of the publisher, the editors and the reviewers. Any product that may be evaluated in this article, or claim that may be made by its manufacturer, is not guaranteed or endorsed by the publisher.

## References

[B1] Becker S. P. Dvorsky M. R. Breaux R. Cusick C. N. Taylor K. P. Langberg J. M. (2021). Prospective examination of adolescent sleep patterns and behaviors before and during COVID-19. Sleep 44, 1–11. 10.1093/sleep/zsab054 33631014 PMC7928571

[B2] Casey B. J. Jones R. M. Hare T. A. (2008). The adolescent brain. Ann. N. Y. Acad. Sci. 1124, 111–126. 10.1196/annals.1440.010 18400927 PMC2475802

[B3] de Vivo L. Bellesi M. Marshall W. Bushong E. A. Ellisman M. H. Tononi G.. (2017). Ultrastructural evidence for synaptic scaling across the wake/sleep cycle. Science 355, 507–510. 10.1126/science.aah5982 28154076 PMC5313037

[B4] Faust T. E. Gunner G. Schafer D. P. (2021). Mechanisms governing activity-dependent synaptic pruning in the developing mammalian CNS. Nat. Rev. Neurosci. 22, 657–673. 10.1038/s41583-021-00507-y 34545240 PMC8541743

[B5] Gariepy G. Danna S. Gobina I. Rasmussen M. Gaspar de Matos M. Tynjala J.. (2020). How are adolescents sleeping? Adolescent sleep patterns and sociodemographic differences in 24 European and North American countries. J. Adolesc. Health 66, S81–S88. 10.1016/j.jadohealth.2020.03.013 32446613

[B6] Hirshkowitz M. Whiton K. Albert S. M. Alessi C. Bruni O. DonCarlos L.. (2015). National Sleep Foundation's sleep time duration recommendations: methodology and results summary. Sleep Health 1, 40–43. 10.1016/j.sleh.2014.12.010 29073412

[B7] Ke M.-T. Nakai Y. Fujimoto S. Takayama R. Yoshida S. Kitajima T. S.. (2016). Super-resolution mapping of neuronal circuitry with an index-optimized clearing agent. Cell Rep. 14, 2718–2732. 10.1016/j.celrep.2016.02.057 26972009

[B8] Li W. Ma L. Yang G. Gan W. B. (2017). REM sleep selectively prunes and maintains new synapses in development and learning. Nat. Neurosci. 20, 427–437. 10.1038/nn.4479 28092659 PMC5535798

[B9] Maret S. Faraguna U. Nelson A. B. Cirelli C. Tononi G. (2011). Sleep and waking modulate spine turnover in the adolescent mouse cortex. Nat. Neurosci. 14, 1418–1420. 10.1038/nn.2934 21983682 PMC3203346

[B10] Moyer C. E. Zuo Y. (2018). Cortical dendritic spine development and plasticity: insights from *in vivo* imaging. Curr. Opin. Neurobiol. 53, 76–82. 10.1016/j.conb.2018.06.002 29936406

[B11] Nagai H. de Vivo L. Marshall W. Tononi G. Cirelli C. (2021). Effects of severe sleep disruption on the synaptic ultrastructure of young mice. eNeuro 8, 1–12. 10.1523/ENEURO.0077-21.2021 34193511 PMC8287877

[B12] Spano G. M. Banningh S. W. Marshall W. de Vivo L. Bellesi M. Loschky S. S.. (2019). Sleep deprivation by exposure to novel objects increases synapse density and axon-spine interface in the hippocampal CA1 region of adolescent mice. J. Neurosci. 39, 6613–6625. 10.1523/JNEUROSCI.0380-19.2019 31263066 PMC6703893

[B13] Wheaton A. G. Claussen A. H. (2021). Short sleep duration among infants, children, and adolescents aged 4 months-17 years - United States, 2016-2018. MMWR Morb. Mortal. Wkly. Rep. 70, 1315–1321. 10.15585/mmwr.mm7038a1 34555000 PMC8459893

[B14] Yang G. Lai C. S. Cichon J. Ma L. Li W. Gan W. B. (2014). Sleep promotes branch-specific formation of dendritic spines after learning. Science 344, 1173–1178. 10.1126/science.1249098 24904169 PMC4447313

[B15] Zhou Y. Lai C. S. W. Bai Y. Li W. Zhao R. Yang G.. (2020). REM sleep promotes experience-dependent dendritic spine elimination in the mouse cortex. Nat. Commun. 11, 4819. 10.1038/s41467-020-18592-5 32968048 PMC7511313

